# The Evolution of Lateralized Brain Circuits

**DOI:** 10.3389/fpsyg.2017.01021

**Published:** 2017-06-16

**Authors:** Michael C. Corballis

**Affiliations:** School of Psychology, University of Auckland,Auckland, New Zealand

**Keywords:** brain asymmetry, evolution, handedness, gesture recognition, mirror neuron system

## Abstract

In the vast clade of animals known as the bilateria, cerebral and behavioral asymmetries emerge against the backdrop of bilateral symmetry, with a functional trade-off between the two. Asymmetries can lead to more efficient processing and packaging of internal structures, but at the expense of efficient adaptation to a natural world without systematic left-right bias. Asymmetries may arise through the fissioning of ancestral structures that are largely symmetrical, creating new circuits. In humans these may include asymmetrical adaptations to language and manufacture, and as one or other hemisphere gains dominance for functions that were previously represented bilaterally. This is best illustrated in the evolution of such functions as language and tool manufacture in humans, which may derive from the mirror-neuron system in primates, but similar principles probably apply to the many other asymmetries now evident in a wide range of animals. Asymmetries arise in largely independent manner with multi-genetic sources, rather than as a single over-riding principle.

## Introduction

Part of the reason for the fascination with handedness and cerebral asymmetry is that they seem to arise from a system that is for the most part structurally symmetrical, suggesting the operation of some non-material force—and perhaps even encouraging a Cartesian notion of mind over matter (Corballis, 1980). Nevertheless lateral asymmetries can scarcely be understood or even defined except in relation to symmetry. Humans belong to the vast clade of animals known as the *bilateria*, going back some 550 million years, and characterized by near symmetry about the midsagittal plane. This bilateral symmetry makes us almost indistinguishable from our reflection in the mirror, and may be an adaptation to the fact that for freely moving animals, the natural world is essentially indifferent with respect to left and right. So it is that we have limbs and sense organs arranged in bilateral pairs, and even the brain is more obviously symmetrical than it is asymmetrical. You would be hard-pressed to decide whether a picture of the brain is normal or mirror-reversed, although there are a few small give-away signs.

It is against this fundamentally symmetrical plan that asymmetries sometimes arise, and are of interest. Structural asymmetries are especially evident in the way internal organs are located, with the heart, stomach, and spleen displaced to the left, the liver and gall bladder to the right. Some asymmetries, such as the asymmetrical gallop of a horse, or human preference for one or other hand, are more apparent from function than from structure. This also seems to be true of the human brain, which functions in well-documented asymmetrical ways that are all the more remarkable given its apparent anatomical symmetry.

The pressure toward asymmetry may have to do, at least in part, with packaging and efficiency, especially in internal structures that are largely independent of external constraints. It would simply be inefficient to pack a suitcase while retaining perfect symmetry of its contents; rather, you fit the contents in to make optimal use of the space. Similarly, an automobile retains external symmetry for efficient movement and maneuverability, while its internal parts are asymmetrically organized. Sheer efficiency may therefore have guided the placement of internal bodily organs such as the stomach, which processes food regardless of the manner of its arrival—or its departure. The heart, too, functions internally and is asymmetrical, but retains a degree of symmetry because it must pump blood to both sides of the body.

There is greater pressure for the retention of symmetry in the brain than in the internal organs of the body, because it is involved in coordination of symmetrical actions such as walking or swimming, and the processing of input from symmetrical sense organs. As the brain increases in size and complexity, though, there would be increased demand for asymmetrical packaging, and this pressure would be enhanced by constraints on the size of the skull. This is especially true of bipedal animals, since the demands of upright walking constrains the size of the birth canal, which in turn restricts the size of the head. These constraints conflict with heightened demands for cognitive processing, especially in animals such as humans where survival depends on complex social interactions and the manufacture of tools and habitable environments. In humans, these competing pressures create what has been termed the “obstetrical dilemma,” a hypothesis to explain why childbirth is so difficult, and leads to dangerously early birth normally requiring assistance (Washburn, 1960)—yet we need large brains to cope with the complexities of our lives on the planet. The pressure for larger brains in a constrained skull can also explain why the human brain is exceptionally wrinkled and folded, like an old automobile crushed for infill. The same conflict might also explain why asymmetry seems especially pronounced in the human brain, since reducing redundancy and duplication makes better use of the restricted brain space.

## The Evolutionary Trade-Off

The trade-off between symmetry and asymmetry is well illustrated, at least in humans, by the hands, and was perhaps a consequence of bipedalism, which freed the hands from involvement in locomotion. The programming of complex actions is most efficiently achieved by an asymmetrical system in the brain rather than one duplicated between hemispheres, yet equal division between the hands is adaptive in simple spatial activities like reaching or plucking, and even in locomotory activities such as swimming. An interesting example is provided by Watson and Kimura (1989), who found that the two hands were equally adept at blocking fast-moving missiles (table-tennis balls), but one hand was much more adept at throwing them than the other. Athletes involved in sports like cricket generally catch well with either hand but throw almost exclusively with just one hand. Activities involving cooperation between the hands, like unscrewing a lid or hammering a nail, also lead to different specializations. As a fairly general rule one hand, usually the left, is used for holding and the other for operating (Bruner, 1968).

Other bipedal species similarly prefer one or other hand in manipulation or in bringing food to the mouth. These include some species of kangaroo (Giljov et al., 2015), which are predominantly left-handed in feeding, and some species of parrot also preferentially use the left foot when picking up bits of food while perching on the right foot (Rogers, 1980; Friedman and Davis, 1938). Some 65–70 percent great apes favor the right hand in various tasks (e.g., Hopkins et al., 2011), with the possible exception of orangutans (Rogers and Kaplan, 1996), but the incidence is lower than that in humans, which stands at around 90 percent. Cerebral asymmetry itself is pervasive in the animal kingdom (Rogers et al., 2013). A general left-hemispheric bias for action dynamics exists in many species, including marine animals and some primates (MacNeilage, 2013). Conversely, a right-hemisphere dominance for emotion seems to be present in all primates so far investigated, suggesting an evolutionary continuity going back at least 30 to 40 million years (Lindell, 2013).

The sense of a trade-off is also suggested by the fact that cerebral and behavioral asymmetries are seldom if ever universal, unlike asymmetries of the internal organs in which the vast majority of individuals show the same asymmetries. Where a given direction of asymmetry is the norm, the proportion of individuals exhibiting the asymmetry lies within the range of about 65–90 percent—a range that seems to apply across the animal kingdom (Ghirlanda and Vallortigara, 2004), with human handedness and cerebral asymmetry at the top of the range. In contrast, the asymmetries of the internal organs are remarkably consistent, with only about one in 10,000 people showing reversal, a condition known as situs inversus (Torgersen, 1950). In the brain, the relative demands of symmetry and asymmetry may therefore be labile, and there may even be population-level advantages in variation. Perhaps the inclusion of a minority of left-handers led to an advantage in warfare or in some sports, but only so long as they remained a minority. There is some evidence that mixed handers are more creative than right- or left-handers handers (Shobe et al., 2009), suggesting that in some endeavors bilaterality may outweigh asymmetry.

Cerebral asymmetry for language is often linked to handedness. For example, Bruner (1968) suggested that the functional difference between the hands could be extrapolated to the cerebral hemispheres, with the right hemisphere holding the context while the left provides the operation, the actual output. More generally, the link between handedness and brain asymmetry may have come about in the evolution of complex manual activities such as the manufacture and use of tools, or more directly through gestural communication itself. Indeed there are some compelling reasons to suppose that language evolved from manual gestures rather than from primate calls (e.g., Hewes, 1973; Corballis, 2002). For example, it has proven virtually impossible to teach great apes anything resembling vocal language, but gestural forms of communication with language-like properties seems to come about quite naturally in chimpanzees, bonobos, and gorillas (Savage-Rumbaugh et al., 1998; Patterson and Gordon, 2001), and is evident in the activities of chimpanzees in the wild (Hobaiter and Byrne, 2011). Again, signed languages are purely gestural, with no functional acoustic component, yet carry all the hallmarks of true language (Emmorey, 2002).

## Multiple Circuits, Multiple Genes

Nevertheless handedness itself is actually rather poorly correlated with cerebral asymmetry for language. Some 95–99 percent of right-handers are left-hemispheric for language, but so are some 70–80 percent of left-handers (Corballis et al., 2012). Different aspects of hemispheric asymmetry are also poorly correlated; one study, for example, shows zero correlation between left-hemispheric dominance for language and right-hemispheric dominance for spatial attention (Badzakova-Trajkov et al., 2010). In another study of brain activity recorded in participants at rest, factor analysis of asymmetries at different sites indicated four independent lateralized networks, two favoring the left hemisphere and two the right (Liu et al., 2009). Such findings suggest that cerebral asymmetry is not due to some all-encompassing gradient, but depends on multiple influences. Indeed, attempts to locate a single laterality gene have largely failed, and it has been suggested that as many as 40 different genes may be involved (McManus et al., 2009).

Factor analyses of task-evoked brain activity also suggest independent circuits. In one study fMRI responses to word generation, processing of faces making emotional expressions, and the landmark test (a measure of spatial attention) yielded three orthogonal factors (Badzakova-Trajkov et al., 2016). One was linked to the language task and represented a left-hemispheric circuit including the pars opercularis and the pars triangularis (together comprising Broca’s area), and the inferior and superior parietal lobules. Another was linked to spatial attention, with right-hemispheric activation predominantly in the pars opercularis, the inferior and superior parietal lobules, and the supramarginal gyrus. The third was also a right-hemispheric circuit linked to face processing, with activation predominantly in the pars triangularis, the fusiform gyrus, and the middle temporal gyrus.

Independent circuits also seem to exist within the left hemisphere. Gonzalez et al. (2006) found that left-hemispheric specialization for the visual control of action was unrelated to handedness, while Króliczak and Frey (2009) identified a circuit concerned with the planning of pantomimes and intransitive gestures, also shown to be independent of handedness (Króliczak et al., 2016). There may be a closer relation, though, between pantomime and language. Vingerhoets et al. (2013) compared samples of those with typical and atypical language dominance, and found strong correlations between brain asymmetry for word generation and for pantomiming tool use. Eighty percent of the participants in each group were left-handed, leaving some question as to whether the relation would hold among right-handers. In a sample of right-handers, Xu et al. (2009) found that spoken language and observation of symbolic gestures, some of which included pantomimes of simple tool use, activated a common left-lateralized network.

Again, though, factor analysis opens the possibility of a more comprehensive account. In one study, both right- and left-handed participants performed simple acts of language production and comprehension, along with observations of action, and factor analysis of laterality measures produced three orthogonal factors, suggesting the existence of three independent networks (Häberling et al., 2016). One was clearly language related, loading on activity in language areas when the participants undertook either of the language tasks. Another, loading on parietal and frontal areas, was activated by observation of actions and was strongly associated with handedness. The third involved frontal and temporal areas partly overlapping with the language circuit, although uncorrelated with it. This circuit was also associated with action observation, but was independent of the handedness circuit. It was the least lateralized and may well be the residue of the original mirror system, dedicated to simple acts such as grasping and reaching (Marangon et al., 2015), but perhaps elaborated to include hand-independent aspects of pantomime (Króliczak and Frey, 2009).

## How Lateralized Circuits Evolve

It is unlikely that new circuits in the brain emerge *de novo*, but are formed from ancestral systems. This can occur in several ways: through expansion and fissioning of an ancestral system into separate systems, through copying and differential modification of an existing circuits, or sometimes through modified circuits fusing to create new functions. These processes in turn can involve the splitting of genes, rather than the emergence of new genes (Oakley and Rivera, 2008). The evolution of new and more specialized circuits may also have increased pressure to lateralization, enabling more efficient packaging and less redundancy and competition. Such pressure may have been especially intense in hominin evolution, as our forebears adapted to increased social and environmental complexity.

In this last example given above, the three circuits may well have derived from the primate mirror system, which responds both when a monkey makes an intentional movement such as grasping a piece of food, and when it observes another individual making the same movement (Rizzolatti and Sinigaglia, 2010). This ancestral system seems to provide an ideal platform for the evolution of language, since it relates the perception of action to its production, and indeed can be taken as further support for the idea that language evolved from manual gestures (Rizzolatti and Arbib, 1998; Corballis, 2002). The mirror property also provides the basis for mutual understanding between speaker and listener (or signer and watcher), an understanding that goes beyond the words themselves and is indeed necessary for effective communication (Sperber and Wilson, 2002). In that respect, language has been characterized as “underdetermined” (Scott-Phillips, 2015).

Within the left hemisphere, then, the language circuit may have fissioned from the ancestral mirror system, and may have been the first new circuit to form, since it was the most lateralized of the three. The circuit linked to handedness may have split off as an adaptation to the use and manufacture of tools, in which handedness is most strongly expressed. And as suggested above, the third and least lateralized circuit, which was independent of handedness, may be the residue of the ancestral mirror system.

A similar fissioning may explain the lateralized representation of reading. Behrmann and Plaut (2015) document evidence that the fusiform gyrus in the primate brain is specialized for face recognition. In humans, the emergence of literacy resulted in a split into a right-hemispheric system for face recognition and a left-hemispheric one for the recognition of printed words, at least among people who have learned to read (literacy is still not universal). This would also have created the asymmetry required for mirror-image discrimination, as in the distinct recognition of letters like *b* and *d* or words like *was* and *saw*. Dehaene and Cohen (2011) describe this process as the “recycling” of cortical territory, originally designated for object and face recognition, for the recognition of spoken words—and Dehaene et al. (2010) suggest that face recognition may suffer as a consequence. It is perhaps not so much a question of recycling, though, as one of the invasion of cortical territory initially dedicated to one function by a related but more specialized function.

This complementarity probably goes beyond the fusiform area. In the analysis by Badzakova-Trajkov et al. (2016), activity of Broca’s area on the left in response to word generation was strongly correlated with activity on its right homolog on the right in response to the processing of videos of facial expressions. This complementarity was partitioned within Broca’s area, with activation on the left stronger in the pars opercularis and that on the right stronger in the pars triangularis. Some asymmetries, then, probably arise as a secondary consequence of an asymmetry emerging in one hemisphere, so the other hemisphere assumes dominance over a function that was previously bilateral.

## Conclusion

Although this scenario suggests that complementarity can arise in the emergence of asymmetries, it does not support the global view of the so-called dual brain, in which each cerebral hemisphere is assumed to represent complementary but global aspects of human cognition, variously characterized as linear, analytical and rational on the left, and divergent, holistic and intuitive on the right (e.g., Ornstein, 1972). This view has persisted to a remarkable degree in modern scholarship (e.g., McGilchrist, 2009) as well as in folklore, and concepts of “left-brain” and “right-brain” thinking are even listed in modern dictionaries. The American Heritage^®^ Dictionary of the English Language (2013), for example, offers the following definitions:

**Left-brained** *adj*: (1) Having the left brain dominant. (2) Of or relating to the thought processes, such as logic and calculation, generally associated with the left brain. (3) Of or relating to a person whose behavior is dominated by logic, analytical thinking and verbal communication, rather than emotion and creativity.

**Right-brained** *adj***:** (1) Having the right brain dominant. (2) Of or relating to the thought processes involved in creativity and imagination, generally associated with the right brain. (3) Of or relating to a person whose behavior is dominated by emotion, creativity, intuition, non-verbal communication and global reasoning rather than logic and analysis.

It has become clear that cerebral asymmetries are more complex and multidimensional, both in terms of their circuitry and their genetic underpinnings. Moreover, cerebral asymmetries are never absolute; even in a strongly left-lateralized function such as language, the right hemisphere makes a significant contribution (e.g., Tailby et al., 2017), and in some individuals representation is bilateral or even predominantly right-hemispheric (Corballis et al., 2012). This suggests a more exacting approach to cerebral asymmetries, and one that takes into account its likely evolutionary precursors.

## Future Directions

Much of the argument of this article is based on the discovery and analysis of lateralized circuits in the human brain, so that conclusions as to their evolution is largely speculative, or loosely based on reverse engineering to animal behavior and physiology. To gain a better appreciation of the evolutionary sequence, future research should be directed more closely to our more recent non-human forebears. Our closest living non-human relatives are chimpanzees and bonobos, with common ancestry among the three species going back some six million years. Over that period, there may have been as many as 20 distinct species of hominin (Wood, 2002), and with the exception of our own fortunate species all are extinct, so we only have fossil evidence as to any evolutionary sequence. *Homo sapiens* is thought to have emerged as a separate species some 200,000 years ago, which is about one thirtieth of the interval from the common ancestry with the great apes, and there is still uncertainty as to whether the transition was punctuational or gradual (Stringer, 2016). According to some, such as Chomsky (2010), language emerged *de novo* uniquely in humans within the past 100,000 years, well after our species emerged—a view that denies evolutionary precursors.

This view, though, is increasingly disputed (Corballis, 2017). We share a common ancestry with the Neanderthals going back some 500,000 years, with a degree of interbreeding, leading some to propose that these large-brained ancestors were cognitively very similar to our own species and probably possessed language (e.g., Dediu and Levinson, 2013; Johansson, 2013). Evidence from their tools also strongly indicates that the majority were right-handed (Uomini, 2011). Further studies of Neanderthals and the closely related Denisovans, especially now that their DNA has been extracted, may eventually bear on how cerebral asymmetry, and indeed language itself, evolved. Morgan et al. (2015) take us back even further, arguing from the manner in which people can be taught Oldowan tool-making technology that language and tool-making must have co-evolved over the past 2.5 million years.

As noted earlier, some great apes, including chimpanzees, show species-level preference for the right hand, albeit less marked, though, than in humans, and there are now techniques for adapting structural MRI for use with chimpanzees. In one recent study, Hopkins et al. (2017) report that skill in a tool-using task designed to simulate termite fishing is associated with increased leftward lateralization of the homolog of Broca’s area and of the hand area of the precentral gyrus. This suggests that the relations between handedness, tool use, and language itself as documented in this article may have evolutionary roots even earlier than the separation of the hominins from the line leading to modern great apes. Another recent study reveals that even capuchins make and use stone tools (Wasserman and Thompson, 2017).

Lateralized circuits seem to characterize such distinctively human attributes as language, the use and manufacture of tools, and social engagement, and further attention to their origins in our hominin and primate forebears will be needed to fully test the account give in this article.

## Author Contributions

The author confirms being the sole contributor of this work and approved it for publication.

## Conflict of Interest Statement

The author declares that the research was conducted in the absence of any commercial or financial relationships that could be construed as a potential conflict of interest.

## References

[B1] Badzakova-Trajkov G. Corballis M. C. Häberling I. S. (2016). Complementarity or independence of hemispheric specializations? A brief review. *Neuropsychologia* 93 386–393. 10.1016/j.neuropsychologia.2015.12.018 26712091

[B2] Badzakova-Trajkov G. Häberling I. S. Roberts R. P. Corballis M. C. (2010). Cerebral asymmetries: complementary and independent processes. *PLoS ONE* 5:e9682 10.1371/journal.pone.0009682 PMC2837380 20300635

[B3] Behrmann M. Plaut D. C. (2015). A vision of graded hemispheric specialization. *Ann. N. Y. Acad. Sci.* 1359 30–46. 10.1111/nyas.12833 26199998 PMC13015954

[B4] Bruner J. S. (1968). *Processes of Cognitive Growth: Infancy*. Worcester, MA: Clark University Press.

[B5] Chomsky N. (2010). “Some simple evo devo theses: how true might they be for language?,” in *The Evolution of Human Language*, eds Larson R. K. Déprez V. Yamakido H. (Cambridge: Cambridge University Press), 45–62.

[B6] Corballis M. C. (1980). Laterality and myth. *Am. Psychol.* 35 284–295. 10.1037/0003-066X.35.3.284 7377654

[B7] Corballis M. C. (2002). *From Hand to Mouth: The Origins of Language*. Princeton, NJ: Princeton University Press.

[B8] Corballis M. C. (2017). Language evolution: a changing perspective. *Trends Cogn. Sci.* 27 229–236. 10.1016/j.tics.2017.01.013 28214132

[B9] Corballis M. C. Badzakova-Trajkov G. Häberling I. S. (2012). Right hand, left brain: genetic and evolutionary bases of cerebral asymmetries for language and manual action. *WIRES Cogn. Sci.* 3 1–17. 10.1002/wcs.158 26302469

[B10] Dediu D. Levinson S. C. (2013). On the antiquity of language: the reinterpretation of Neandertal linguistic capacities and its consequences. *Front. Psychol.* 4:397 10.3389/fpsyg.2013.00397 PMC3701805 23847571

[B11] Dehaene D. Pegado F. Braga L. W. Ventura P. Filho G. N. Jobert A. (2010). How learning to read changes the cortical networks for vision and language. *Science* 330 1359–1364. 10.1126/science.1194140 21071632

[B12] Dehaene S. Cohen L. (2011). The unique role of the visual word form area in reading. *Trends Cogn. Sci.* 15 255–261. 10.1016/j.tics.2011.04.003 21592844

[B13] Emmorey K. (2002). *Language, Cognition, and Brain: Insights from Sign Language Research*. Hillsdale, NJ: Erlbaum.

[B14] Friedman H. Davis M. (1938). “Left-handedness” in parrots. *Auk* 35 478–480. 10.2307/4078415

[B15] Ghirlanda S. Vallortigara G. (2004). The evolution of brain lateralization: a game-theoretical analysis of population structure. *Proc. Roy. Soc. B* 271 853–857. 10.1098/rspb.2003.2669 PMC1691668 15255105

[B16] Giljov A. Karenina K. Ingram J. Malashichev Y. (2015). Parallel emergence of true handedness in the evolution of marsupials and placentals. *Curr. Biol.* 14 1878–1884. 10.1016/j.cub.2015.05.043 26096972

[B17] Gonzalez C. L. R. Ganel T. Goodale M. A. (2006). Hemispheric specialization for the visual control of action is independent of handedness. *J. Neurophysiol.* 95 3496–3501. 10.1152/jn.01187.2005 16495359

[B18] Häberling I. S. Corballis P. M. Corballis M. C. (2016). Language, gesture, and handedness: evidence for independent lateralized networks. *Cortex* 82 72–85. 10.1016/j.cortex.2016.06.003 27367793

[B19] Hewes G. W. (1973). Primate communication and the gestural origins of language. *Curr. Anthropol.* 14 5–24. 10.1086/201401

[B20] Hobaiter C. Byrne R. W. (2011). Serial gesturing by wild chimpanzees: its nature and function for communication. *Anim. Cogn.* 14 827–838. 10.1007/s10071-011-0416-3 21562816

[B21] Hopkins W. D. Meguerditchian A. Coulon O. Misiura M. Pope S. Mareno M. C. (2017). Motor skill for tool-use is associated with asymmetries in Broca’s area and the motor hand area of the precentral gyrus in chimpanzees (Pan troglodytes). *Behav. Brain Res.* 318 71–81. 10.1016/j.bbr.2016.10.048 27816558 PMC5459306

[B22] Hopkins W. D. Phillips K. A. Bania A. Calcutt S. E. Gardner M. Russell J. (2011). Hand preferences for coordinated bimanual actions in 777 great apes: implications for the evolution of handedness in hominins. *J. Hum. Evol.* 60 605–611. 10.1016/j.jhevol.2010.12.008 21334723 PMC3068228

[B23] Johansson S. (2013). The talking Neanderthals: what do fossils, genetics, and archeology say? *Biolinguistics* 7 35–74.

[B24] Króliczak G. Frey S. H. (2009). A common network in the left cerebral hemisphere represents planning of tool use pantomimes and familiar intransitive gestures at the hand-independent level. *Cereb. Cortex* 19 2396–2410. 10.1093/cercor/bhn261 19181695 PMC2742597

[B25] Króliczak G. Piper B. J. Frey S. H. (2016). Specialization of the left supramarginal gyrus for hand-independent praxis representation is not related to hand dominance. *Neuropsychologia* 93 501–512. 10.1016/j.neuropsychologia.2016.03.023 27020138 PMC5036996

[B26] Lindell A. K. (2013). Continuities in emotion lateralization in human and nonhuman primates. *Front. Hum. Neurosci.* 7:464 10.3389/fnhum.2013.00464 PMC3737467 23964230

[B27] Liu H. Stufflebeam S. M. Sepulcre J. Hedden T. Buckner R. (2009). Evidence from intrinsic activity that asymmetry of the human brain is controlled by multiple factors. *Proc. Natl. Acad. Sci. U.S.A.* 106 20499–20503. 10.1073/pnas.0908073106 19918055 PMC2777963

[B28] MacNeilage P. F. (2013). Vertebrate whole-body-action asymmetries and the evolution of right handedness: a comparison between humans and marine mammals. *Dev. Psychobiol.* 55 577–587. 10.1002/dev.21114 23913760

[B29] Marangon M. Kubiak A. Króliczak G. (2015). Haptically guided grasping: fMRI shows right-hemisphere parietal stimulus encoding, and bilateral dorso-ventral parietal gradients of object- and action-related processing during grasp execution. *Front. Hum. Neurosci.* 9:691 10.3389/fnhum.2015.00691 PMC4700263 26779002

[B30] McGilchrist I. (2009). *The Master and his Emissary: The Divided Brain and the Making of the Western World*. New Haven, CT: Yale University Press.

[B31] McManus I. C. Davison A. Armour J. A. L. (2009). Multilocus genetic models of handedness closely resemble single-locus models in explaining family data and are compatible with genome-wide association studies. *Ann. N. Y. Acad. Sci.* 1288 48–58. 10.1111/nyas.12102 PMC4298034 23631511

[B32] Morgan T. J. H. Uomini N. T. Rendell L. E. Chouinard-Thuly L. Street S. E. Lewis H. M. (2015). Experimental evidence for the co-evolution of hominin tool-making teaching and language. *Nat. Commun.* 6:6029 10.1038/ncomms7029 PMC4338549 25585382

[B33] Oakley T. H. Rivera A. S. (2008). Genomics and the evolutionary origins of nervous system complexity. *Curr. Opin. Genet. Dev.* 18 479–492. 10.1016/j.gde.2008.12.002 19152785

[B34] Ornstein R. E. (1972). *The Psychology of Consciousness.* San Francisco, CA: Freeman.

[B35] Patterson F. G. P. Gordon W. (2001). “Twenty-seven years of project Koko and Michael,” in *All Apes Great and Small: African Apes* Vol. 1 eds Galdikas V. Briggs N. Erickson Sheeran L. K. Goodall J. (New York, NY: Kluver), 165–176.

[B36] Rizzolatti G. Arbib M. A. (1998). Language within our grasp. *Trends Neurosci.* 21 188–194. 10.1016/S0166-2236(98)01260-0 9610880

[B37] Rizzolatti G. Sinigaglia C. (2010). The functional role of the parieto-frontal mirror circuit: interpretations and misinterpretations. *Nat. Rev. Neurosci.* 11 264–274. 10.1038/nrn2805 20216547

[B38] Rogers L. J. (1980). Lateralization in the avian brain. *Bird Behav.* 2 1–12. 10.3727/015613880791573835

[B39] Rogers L. J. Kaplan G. (1996). Hand preferences and other lateral biases in rehabilitated orang-utans, *Pongo pygmaeus*. *Anim. Behav.* 51 13–25. 10.1016/j.bbr.2009.11.011

[B40] Rogers L. J. Vallortigara G. Andrew R. J. (2013). *Divided Brains: The Biology and Behavior of Brain Asymmetries.* Cambridge: Cambridge University Press 10.1017/CBO9780511793899

[B41] Savage-Rumbaugh S. Shanker S. G. Taylor T. J. (1998). *Apes, Language, and the Human Mind.* New York: Oxford University Press.

[B42] Scott-Phillips T. (2015). *Speaking Our Minds: Why Human Communication is Different, and How Language Evolved to Make it Special.* Basingstoke: Palgrave Macmillan. 10.1007/978-1-137-31273-0

[B43] Shobe E. R. Ross N. M. Fleck J. I. (2009). Influence of handedness and bilateral eye movements on creativity. *Brain Cogn.* 71 204–214. 10.1080/1357650X.2015.1089879 19800726

[B44] Sperber D. Wilson D. (2002). Pragmatics, modularity and mind-reading. *Mind Lang.* 17 3–23. 10.1111/1468-0017.00186

[B45] Stringer C. (2016). The origin and evolution of Homo sapiens. *Philos. Trans. R. Soc. B* 371 20150237 10.1098/rstb.2015.0237 PMC4920294 27298468

[B46] Tailby C. Abbott D. F. Jackson G. D. (2017). The diminishing dominance of the dominant hemisphere: language fMRI in focal epilepsy. *Neuroimage Clin.* 14 141–150. 10.1016/j.nicl.2017.01.011 28180072 PMC5279902

[B47] The American Heritage Dictionary of the English Language (2013). *The American Heritage^®^ Dictionary of the English Language*, 5th Edn. New York, NY: Houghton Mifflin.

[B48] Torgersen J. (1950). Situs inversus, asymmetry, and twinning. *Am. J. Hum. Genet.* 2 361–370. 14837905 PMC1716380

[B49] Uomini N. T. (2011). “Handedness in Neanderthals,” in *Neanderthal Lifeways, Subsistence and Technology*, eds Conard N. J. Richter J. (New York, NY: Springer Publishing).

[B50] Vingerhoets G. Alderweireldt A. S. Vandemaele P. Cai Q. Van der Haegen L. Brysbaert M. (2013). Praxis and language are linked: evidence from co-lateralization in individuals with atypical language dominance. *Cortex* 49 172–183. 10.1016/j.cortex.2011.11.003 22172977

[B51] Washburn S. L. (1960). Tools and human evolution. *Sci. Am.* 203 3–15. 13843002

[B52] Wasserman E. A. Thompson R. K. R. (2017). Capuchin monkeys can make and use stone tools. *Learn. Behav.* 45 103–104. 10.3758/s13420-016-0257-7 28078654 PMC5429872

[B53] Watson N. V. Kimura D. (1989). Right-hand superiority for throwing but not for intercepting. *Neuropsychologia* 27 1399–1414. 10.1016/0028-3932(89)90133-4 2615939

[B54] Wood B. (2002). Hominid revelations from Chad. *Nature* 481 133–135. 10.1038/418133a 12110870

[B55] Xu J. Gannon P. J. Emmorey K. Smith J. F. Braun A. R. (2009). Symbolic gestures and spoken language are processed by a common neural system. *Proc. Natl. Acad. Sci. U.S.A.* 106 20664–20669. 10.1073/pnas.0909197106 19923436 PMC2779203

